# TLR10 Senses HIV-1 Proteins and Significantly Enhances HIV-1 Infection

**DOI:** 10.3389/fimmu.2019.00482

**Published:** 2019-03-15

**Authors:** Bethany M. Henrick, Xiao-Dan Yao, Muhammad Atif Zahoor, Alash'le Abimiku, Sophia Osawe, Kenneth L. Rosenthal

**Affiliations:** ^1^Evolve Biosystems, Davis, CA, United States; ^2^Department of Food Science and Technology, University of Nebraska, Lincoln, NE, United States; ^3^Department of Pathology and Molecular Medicine, McMaster University, Hamilton, ON, Canada; ^4^Institue of Human Virology-Nigeria, Abuja, Nigeria

**Keywords:** human breast milk, HIV-1, gp41, p17, p24, TLR10, IL-8, NF-κBα

## Abstract

Toll-like receptors (TLRs) play a crucial role in innate immunity and provide a first line of host defense against invading pathogens. Of the identified human TLRs, TLR10 remains an orphan receptor whose ligands and functions are poorly understood. In the present study, we sought to evaluate the level of TLR10 expression in breast milk (BM) and explore its potential function in the context of HIV-1 infection. We evaluated HIV-1-infected (Nigerian: *n* = 40) and uninfected (Nigerian: *n* = 27; Canadian: *n* = 15) BM samples for TLR expression (i.e., TLR10, TLR2, and TLR1) and report here that HIV-1-infected BM from Nigerian women showed significantly higher levels of TLR10, TLR1, and TLR2 expression. Moreover, the level of TLR10 expression in HIV-1-infected BM was upregulated by over 100-fold compared to that from uninfected control women. *In vitro* studies using TZMbl cells demonstrated that TLR10 overexpression contributes to higher HIV-1 infection and proviral DNA integration. Conversely, TLR10 inhibition significantly decreased HIV-1 infection. Notably, HIV-1 gp41 was recognized as a TLR10 ligand, leading to the induction of IL-8 and NF-κBα activation. The identification of a TLR10 ligand and its involvement in HIV-1 infection enhances our current understanding of HIV-1 replication and may assist in the development of improved future therapeutic strategies.

## Introduction

Toll-like receptors (TLRs) are the most well-known and researched family of pattern recognition receptors (PRRs) that recognize conserved regions of pathogen-associated molecular patterns (PAMPs) and endogenous danger-associated molecular patterns (DAMPs) responsible for driving cellular activation central to innate immunity. To date, 10 TLRs have been identified in humans, with TLR10 being the only remaining family member without a clearly defined ligand (1, 2); however, a number of ligands, including synthesized TLR2 ligands (i.e., Pam3CSK4 and PamCysPamSK_4_), have been identified as potential TLR10 PAMPs through computational protein modeling (3).

Once believed to be only preferentially expressed on various types of immune cells (4), TLR10 has now been identified in multiple mucosal sites, including the small intestine, fallopian tubes, eye, and stomach (5–8). Interestingly, many of these sites are also highly affected during acute viral infections, including HIV-1; therefore, identifying ligands for TLR10 and defining the mechanisms of host defense following viral infection is of particular interest for the development of novel vaccines and drug therapies.

Immune activation is critical to HIV-1 infection and pathogenesis, leading to increased proinflammatory cytokine production, T cell exhaustion, and the eventual development of opportunistic infections (9); however, our understanding of immunopathogenesis remains incomplete. Historically, research has focused on HIV-1-specific adaptive immune responses, whereas the role of innate immunity has been largely overlooked, despite the fact that it provides the first line of defense and shapes subsequent adaptive responses. Although the identification of HIV-1 PAMPs recognized by PRRs remain poorly elucidated, data indicate that peripheral blood mononuclear cells (PBMCs) and plasmacytoid dendritic cells (pDCs) derived from HIV-1 infected individuals have increased levels of TLR2, TLR3, TLR4, TLR6, TLR7, and TLR8 mRNA at various stages of disease progression (10, 11). In addition, our laboratory has previously investigated the genital epithelium of Kenyan commercial sex workers and found significant modification in TLR expression, which correlates with resistance to HIV-1 (12). Furthermore, we were the first to demonstrate that soluble TLR2 (sTLR2), which is highly prevalent in human breast milk (BM) (13) serves as an innate antiviral factor in BM and significantly inhibits HIV-1 infection and integration *in vitro* (14, 15). We further reported a significant increase in TLR2 expression in BM cells, and that the overexpression of TLR2 in reporter cells greatly enhanced HIV-1 infection *in vitro* (15). We further identified HIV-1-specific structural proteins, p17, p24, and gp41, which serve as PAMPs, leading to significantly increased immunopathogenesis and infection *in vitro* (16).

Given that TLR10 is a homolog of both TLR2 and TLR1, we hypothesized that TLR10 is involved in sensing specific HIV-1 structural proteins, which leads to increased cellular activation and HIV-1 infection. In this study, we report highly significantly increased TLR10 and TLR1 expression in HIV-1-infected human primary BM cells. Additionally, for the first time, TLR10 was found to be involved in innate immune sensing and cellular activation induced by HIV-1, leading to increased infection *in vitro*. Taken together, we provide clear evidence that specific HIV-1 structural proteins trigger TLR10-dependent cellular activation. These findings indicate that TLR10 and its heterodimers, TLR1 and TLR2, play a central role in the innate immune response to HIV-1 infection by sensing viral proteins, leading to increased immunopathogenesis.

## Results

### Healthy Human BM Derived Macrophages and Mammary Epithelial Cells Express TLR10, TLR2, and TLR1

**Figure 1:**
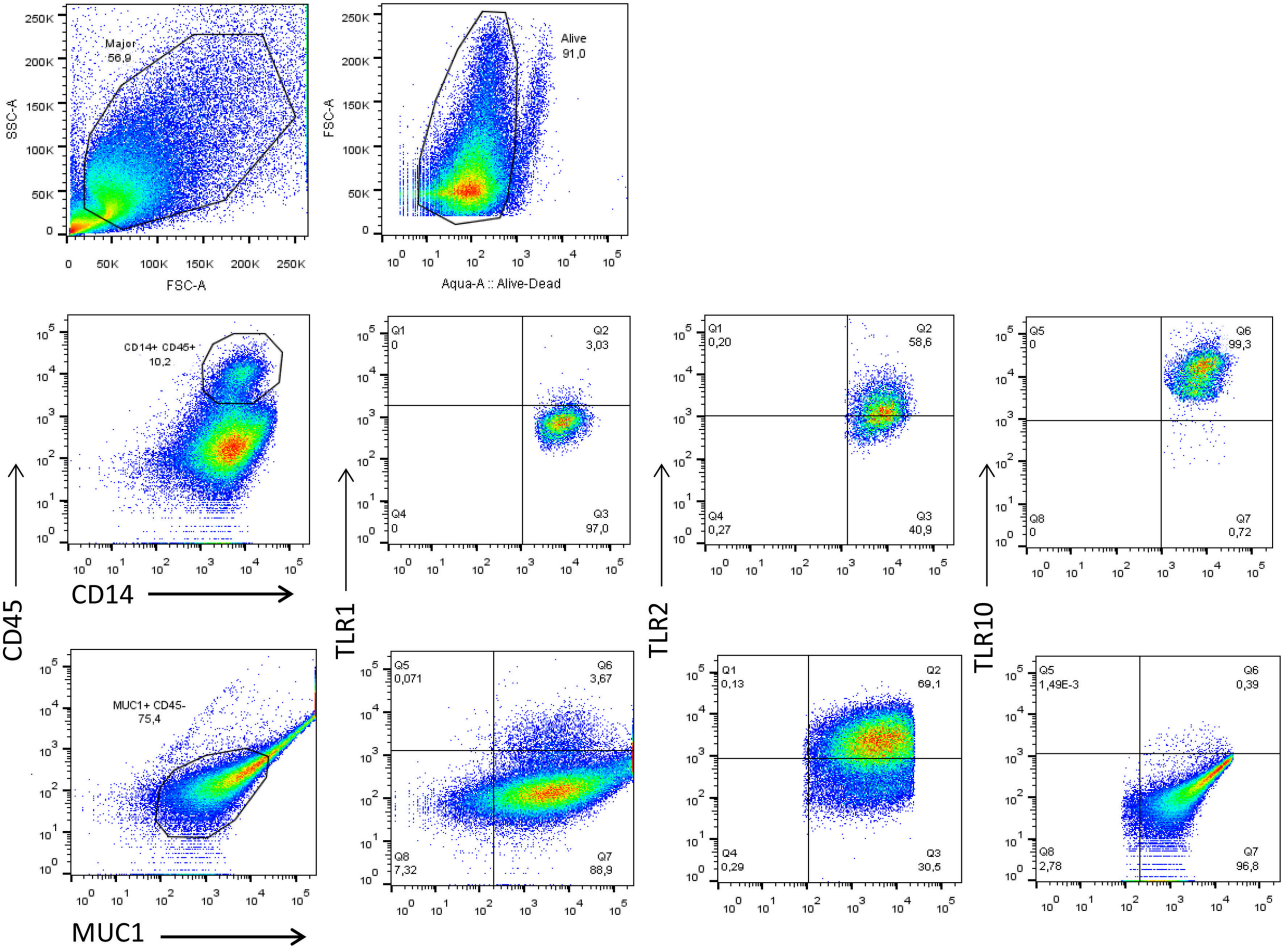
Flow cytometry analyses of TLR1, TLR2, and TLR10 expression in the two major types of primary BM cells obtained from HIV-1 uninfected women at 3 months post-partum. Leucocytes were gated with CD45-PerCP-Cy5.5 and CD14-V450 whereas the epithelial cells were gated with MUC1-PE-Cy7 and CD45-. The antibodies TLR1-APC TLR2-APC and TLR10-PE were separately used to detect TLR1, TLR2, and TLR10 in CD14^+^-CD45^+^-MUC1^−^ and MUC1^+^-CD45^−^ cell types to differentiate the two major constituents of BM. Representative images are shown with TLR1, TLR2, and TLR10 expression depicted as percentages on right corner of each image.

Depending on the stage of lactation, the predominant cell types in BM consist of a variety of leukocytes in colostrum (4 × 10^6^ cells/mL) and mature BM (10^5^-10^6^ cells/mL), as well as mammary epithelial cells (MECs) (16–18). The majority of leukocytes present in BM display an activated phenotype (19) and are comprised primarily of macrophages (55–60%) and neutrophils (30–40%), with the remaining 5–10% composed of lymphocytes (~65% CD8+ T cells, 15% CD4+ T cells, and 20% B cells) (18). Macrophages and MECs are thought to facilitate mother-to-child transmission (MTCT) of HIV-1, and both cell types express several canonical HIV-1 receptors, (e.g., CD4 and CCR5), readily endocytose cell-free HIV-1, and can function as a viral reservoir (20). To explore how TLR expression is linked to HIV-1 infection, we first analyzed the phenotype of the macrophages and MECs present in HIV-1 uninfected human BM using flow cytometry. We found that the majority of cells that were CD14+ and CD45+ (>10%) expressed TLR1 (3.03%), TLR2 (58.6%), and TLR10 (99.3%). Similarly, MUC1+ (75.4%) MECs also expressed TLR1, TLR2, and TLR10 exhibiting 3.67, 69.1, and 0.39%, respectively (Figure 1). Based on our flow cytometry data our findings suggest that BM derived macrophages predominantly express TLR10 which implies that these cells may play an important role in innate immunity against invading pathogens in suckling infants.

### TLR10 and TLR1 Are Highly Expressed in HIV-1 Infected Human Primary BM Cells

**Figure 2:**
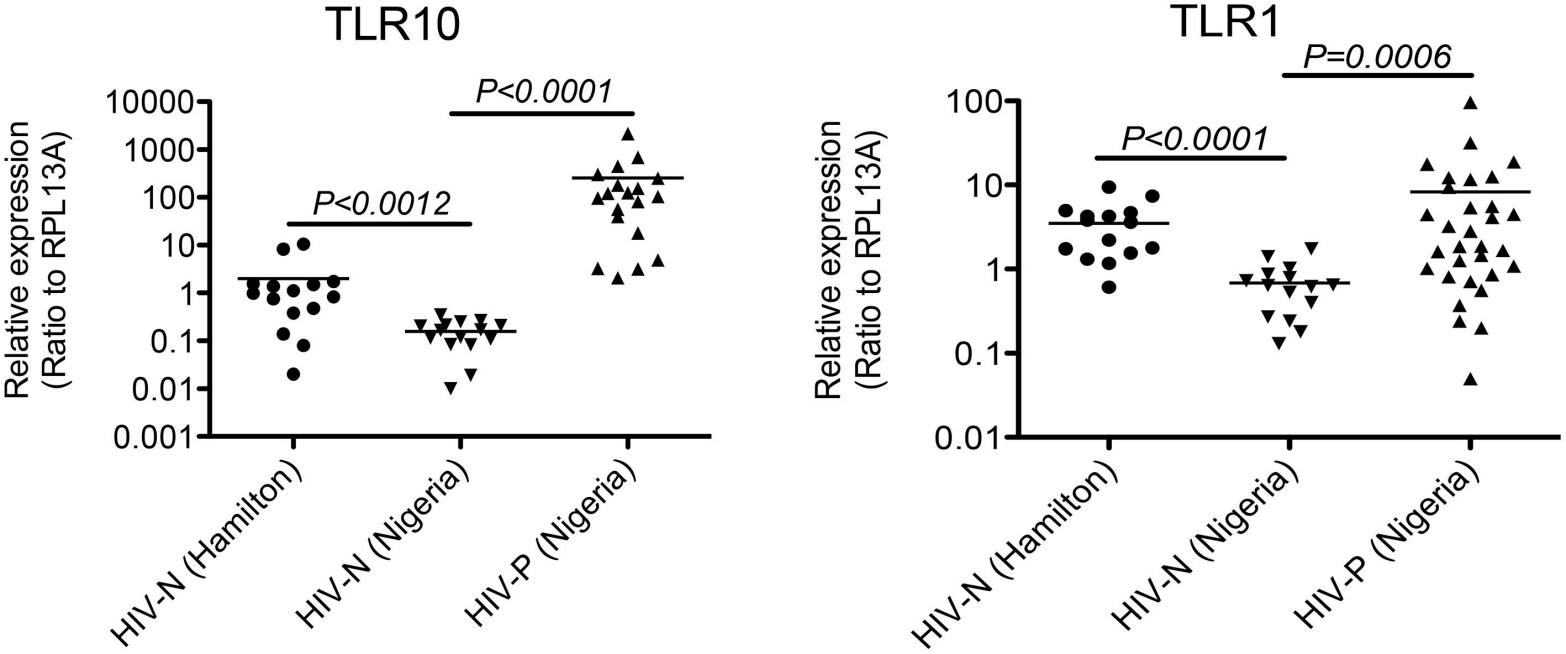
Significantly elevated expression level of TLR1 and TLR10 in HIV-1 infected human primary BM cells. Expression of TLR1 and TLR10 as measured by qRT-PCR with the mRNA extracted from primary BM cells collected from HIV-1 negative Hamilton, Canada women (HIV-N Hamilton) and Nigerian HIV-1 negative (HIV-N) and HIV-1 positive (HIV-P) women. TLR1 expression is shown on right (*p* = 0.0006) whereas TLR10 expression is shown on left (*p* < 0.0001).

The biological relevance of primary BM cells in HIV MTCT remains unclear. BM derived TLRs such as TLR2, TLR3, and TLR5 along with other bioactive molecules are known to play an important role in intestinal protection, inflammatory responses and even microbial recognition (21). Indeed, it has been suggested that certain cytokines such as interferon gamma secreted by primary BM cells reduce the rate of BM transmission of HIV-1 in breastfeeding infants (22, 23); therefore, we next sought to determine the level of TLR10 and TLR1 expression in BM collected from HIV-1 infected women from Nigeria and compared to HIV-1 uninfected women. Both HIV-1 infected as well as uninfected control women were recruited from Plateau State, Nigeria and a series of BM samples from first week to 52 weeks postpartum were collected and shipped to Canada. All HIV-1 positive women were on antiretroviral therapy (ART) and receiving antiretroviral drugs according to the regimen set by Nigerian Government and world Health Organization (WHO) as described (24) We evaluated HIV-1-infected (Nigerian: *n* = 40) and uninfected (Nigerian: *n* = 27; Canadian: *n* = 15) BM samples for the expression of TLR10 and TLR1. Our results clearly demonstrated a highly significant increase in the expression of both TLR1 and TLR10 cDNA in HIV-1-infected compared to uninfected primary BM cells from the same geographical location (Figure 2; *P* = *0.0006* and *P* < *0.0001*, respectively). Specifically, TLR10 expression was ~100-fold higher in HIV-1 infected vs. HIV-1 uninfected primary BM cells. In addition to HIV-1 infected and uninfected women from Nigeria, we also collected BM samples from uninfected healthy women from Hamilton region, Ontario, Canada and compared their TLR1 and TLR10 expression levels with uninfected healthy control women from Nigeria. Interestingly, it was found that TLR1 and TLR10 expression on Nigerian HIV-1 uninfected primary BM cells was significantly lower compared to that on Canadian HIV-1 uninfected primary BM cells thus highlighting the demographic differences. Taken together, these results show that TLRs are highly expressed on primary BM cells, and this expression may be closely associated with an individual's HIV-1 infection and innate immune status.

### The Level of TLR10 Expression Significantly Alters HIV-1 Infection and Integration

**Figure 3:**
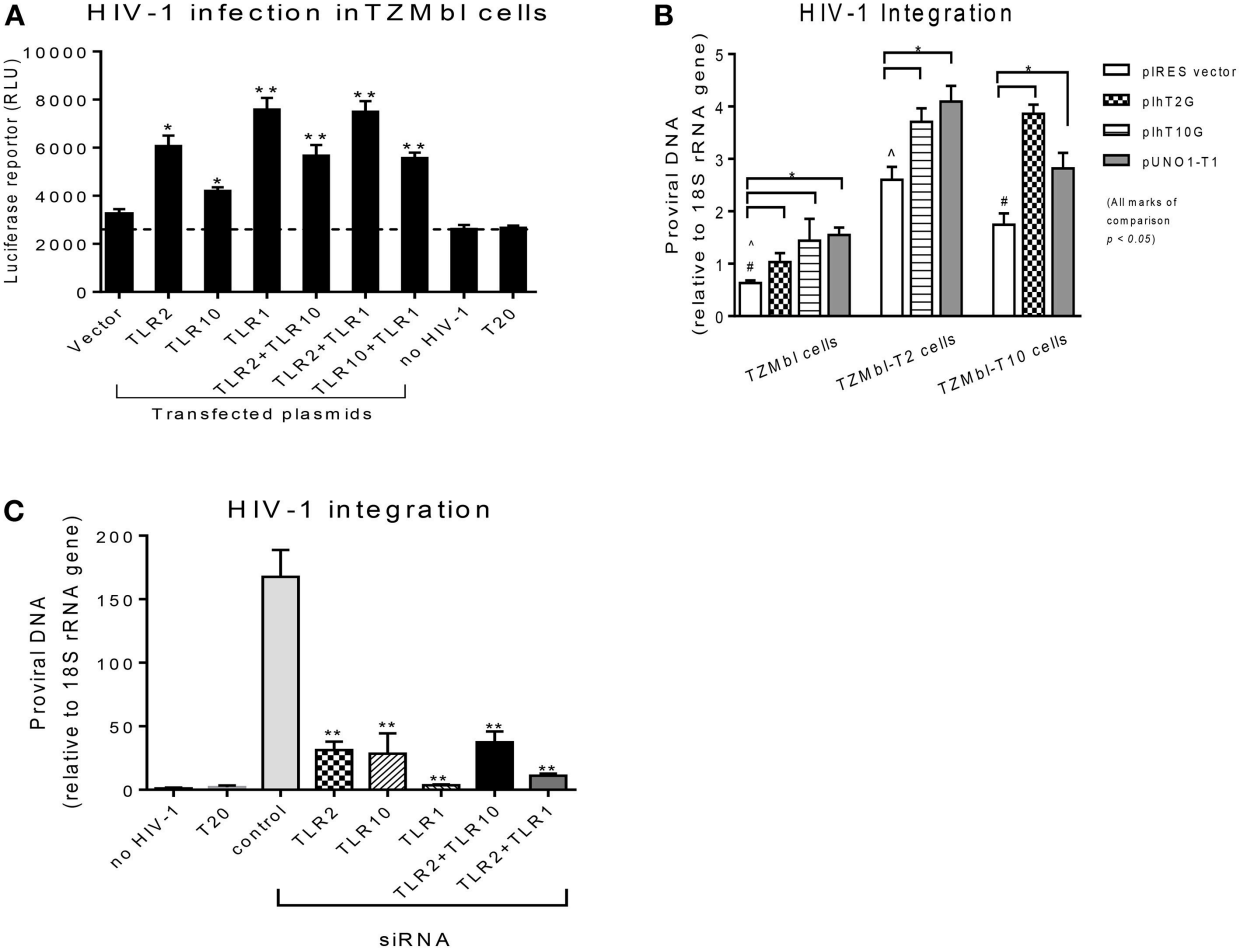
Overexpression or siRNA mediated knockdown of TLR10 significantly alters HIV-1 infection and integration **(A)** HIV-1 infection was significantly enhanced in HIV-1 reporter TZMbl cells transiently overexpressing TLR10 alone and co-transfected with TLR2 or TLR1 expression plasmids by measuring luciferase activity in relative light units (RLU). **(B)** HIV-1 integration was significantly increased in stable TZMbl reporter cells overexpressing TLR10, TLR2, and TLR1. TZMbl, TLR2- stable, and TLR10-stable cells were used for co-transfection with plasmids: empty vector, TLR2, TLR10, and TLR1 vector, TLR10 and TLR1 vector, and TLR2 and TLR1 vector. Proviral DNA (DNA pol) was detected by PCR and normalized to the 18S rRNA gene. **(C)** Proviral DNA was obviously decreased in macrophages with TLR10 knocked down prior to HIV-1 infection. T20: Enfuvirtide, an HIV-1 fusion inhibitor used as a negative control. Data set is representative of three different experiments completed in triplicate (Statistic marks in the plots: ^*^p < *0.05*, ^**^p < *0.01* for Mann Whitney *t*-tests, each group compared to the vector group in A and B, or to the control group in C; pair comparison ∧, # in B, respectively).

Since the extracellular expression of TLR10, TLR1, and TLR2 are innate immune molecules involved in pathogen signaling and are highly expressed on cells in BM (Figures 1, 2) and PBMCs (1, 25) we decided to utilize human mammary epithelial (Michigan Cancer Foundation-10A; MCF-10A) cells and macrophage cell lines (human acute monocytic leukemia; THP-1) for further downstream experiments. MCF-10A is a human non-tumorigenic epithelial cell line with no signs of terminal differentiation and has been used in our previous studies (15). THP-1 is an immortalized monocyte-like cell line derived from the peripheral blood of a childhood case of acute monocytic leukemia (26, 27) and has been utilized previously (28). First we determined whether the expression levels could influence HIV-1 infection *in vitro*. For this purpose, HIV-1 reporter TZMbl cells (29) were transiently transfected alone or in combination with plasmids overexpressing TLR10 and the heterodimers, TLR2 and TLR1 (Figure 3A), followed by HIV-1 infection and measurement of luciferase activity. The results showed a significantly increased rate of HIV-1 infection in TZMbl cells overexpressing TLR10 and heterodimers TLR1 or TLR2 compared to the empty vector alone, uninfected control or a T20 control (Enfuvirtide; HIV-1 fusion inhibitor) (30, 31) (Figure 3A; *P* < 0.05). In addition, HIV-1 infection was significantly elevated in TZMbl cells, which were either co-transfected with TLR1/10 or TLR2/10 compared to the control (Figure 3A; *P* < 0.05).

Next, we determined the extent of HIV-1 infection in TLR10 and TLR2 stable reporter cell lines. TZMbl-T2 and TZMbl-T10 displayed significantly higher levels of proviral DNA compared to TZMbl cells, which served as a control (Figure 3B; *P* < 0.05). Furthermore, stable TZMbl-T2 transiently over-expressing TLR10 or TLR1 also enhanced HIV-1 pol gene expression. Similarly, stable TZMbl-T10 cells transiently over-expressing TLR2 or TLR1 also enhanced proviral pol DNA at 8 h post-infection compared with the empty vector-transient stable cells (Figure 3B; *P* < 0.05).

In order to observe the HIV-1 infection in susceptible cells, macrophages were differentiated from THP-1 cells to determine whether siRNA-mediated knockdown of TLR1, 2 and/or 10 for 2 days prior to CCR5-tropic HIV-1 (BAL strain) exposure for 7 days would affect the HIV-1 infection rate. THP-1 cells were first transfected with siRNAs targeting TRL1, TLR2, or TLR10, and their knockdown was confirmed by Western blot. TLR1 and TLR2 knockdown was complete compared to TLR10 where it was partial (Supplementary Figure 1). After the confirmation of the siRNA-mediated knockdown, HIV-1 integration was determined by PCR, which showed significantly decreased proviral DNA production in the cells that received siRNAs against TLR1, TLR2, TLR10, or combinations of TLR2 with TRL10 or TLR1 compared to the control siRNA (Figure 3C). In addition, since we observed incomplete TLR10 knockdown in THP-1 cells, it is likely that upon complete knockdown of TLR10, HIV-1 integration and proviral DNA production will be impacted. Together, these data indicate that the expression of TLR1, 2 and TLR10 alone or in combination significantly enhances HIV-1 infection and integration *in vitro*. Moreover, based on TLR10 and TLR2 co-transfection or co-depletion through siRNA and effect on HIV-1 infection or integration, TLR10 appears to play a role in HIV-1 infection and integration independent of TLR2 since no additive effect of TLR10 and TLR2 was observed (Figures 3B,C). Therefore, it is highly likely that TLR2 and TLR10 are independently involved in the regulation of HIV-1 infection in a non-heterodimeric form.

### Expression of TLR10, TLR1, and TLR2 Is Up-Regulated by PAMPs and HIV-1

**Figure 4:**
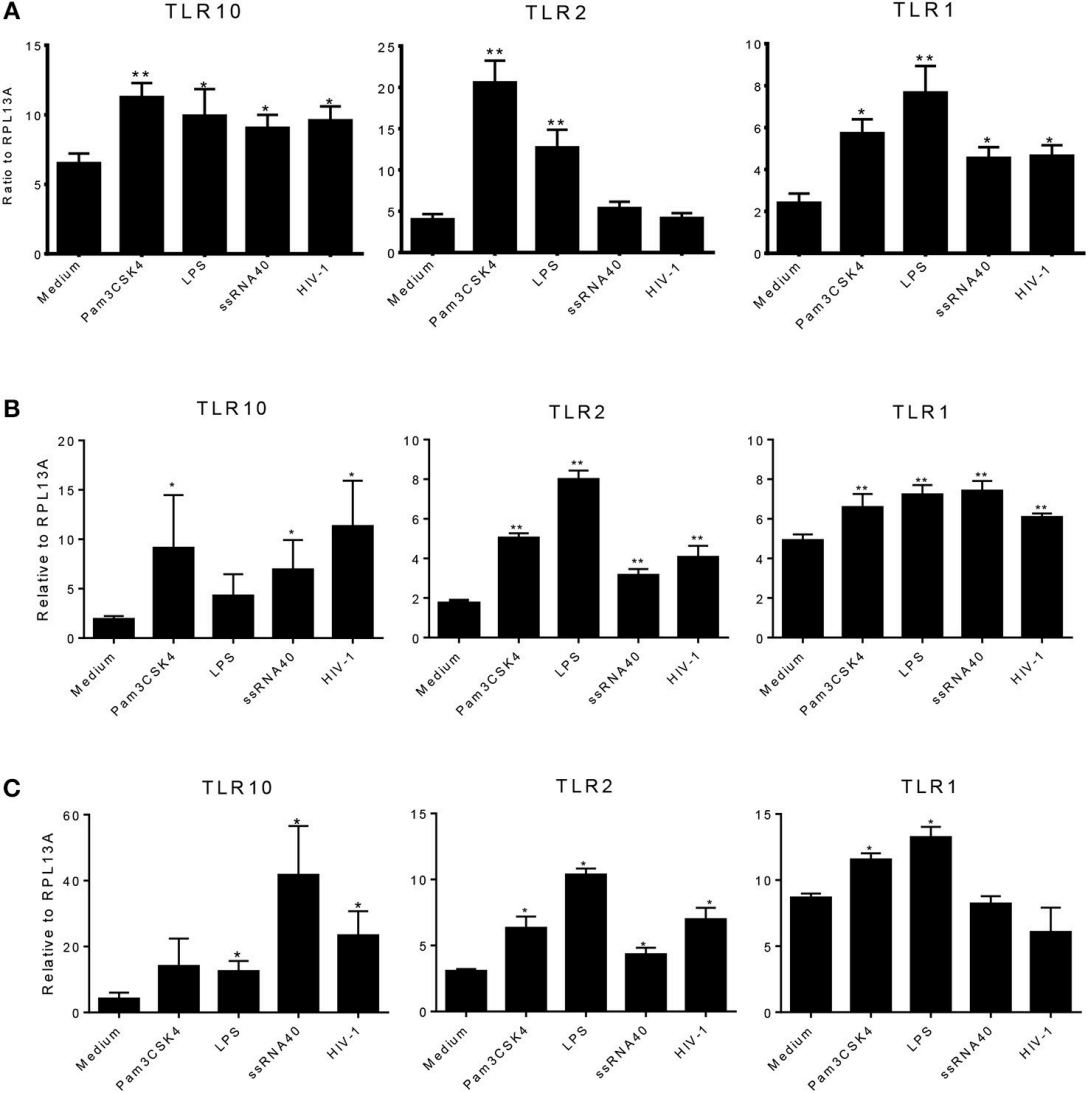
Effects of PAMPs and HIV-1 on the level of TLR expression in cell cultures. **(A)** qRT-PCR of TLR10, TLR2, and TLR1 mRNA from MCF-10A cells treated with TLR2 PAMP Pam3CSK4, TLR4 PAMP LPS, TLR7/TLR8 PAMP ssRNA40, and cell-free HIV-1 BAL. **(B)** q-RT-PCR in THP-1 cells. **(C)** qRT-PCR in primary BM cells. Data set is representative of three different experiments completed in triplicate. Statistic marks: ^*^p < *0.05*, ^**^p < *0.01* for the Mann-Whitney *t*-tests, each group compared to medium-treated group (Medium).

We next determined whether the expression of TLR1, 2, or 10 changes in response to various TLR ligands. MCF-10A, THP-1, and primary BM cells were stimulated with TLR ligands of interest at the following doses: 100 ng/mL Pam3Cys-Ser-(Lys)4 (Pam3CSK4); 10 ng/mL lipopolysaccharide (LPS), ssRNA40 1 μg/mL, and cell-free HIV-1 overnight. Total RNA was isolated from the cells and qRT-PCR was performed. Data showed that compared to medium control, MCF-10A cells exposed to the TLR2/1 ligand, Pam3CSK4, significantly increased the level of TLR1, 2, and 10 expression (Figure 4A). Similarly, exposure to TLR4 ligand, LPS, significantly increased TLR1 and 2 expression (Figure 4A). In contrast, although ssRNA40 showed a significant increase in TLR1 and TLR10, but had no significant effect on TLR2 expression (Figure 4A). Conversely, HIV-1 exposure significantly increased the level of TLR10 expression on MCF-10A cells (Figure 4A) despite the fact that HIV-1 does not productively infect this cell type (20). In THP-1 cells, Pam3CSK4 exposure significantly increased the level of TLR2 and 10 expression, whereas LPS exposure resulted in an increase in TLR1 and 2 expression (Figure 4B). Following exposure to ssRNA40, the expression of TLR1, 2, and 10 was significantly increased. Similarly, HIV-1 exposure significantly increased the level of TLR1, 2, and 10 expression in THP-1 cells (Figure 4B). Primary BM cells exposed to specific PAMPs also exhibited significantly increased levels of TLR1 and TLR2 expression following exposure to Pam3CSK4 and LPS (Figure 4C). Elevated TLR2 expression was also observed after exposure to ssRNA40 and cell-free HIV-1 (Figure 4C). Most importantly, the data indicated that TLR10 was significantly elevated after exposure to ssRNA40 and HIV-1 (Figure 4C). Taken together, these data indicate that specific HIV-1 PAMPs, as well as cell-free HIV-1, significantly increase TLR10, TLR1, and TLR2 expression in primary BM cells.

### HIV-1 Proteins Regulate TLR Expression Levels and Induce IL-8 Secretion

**Figure 5:**
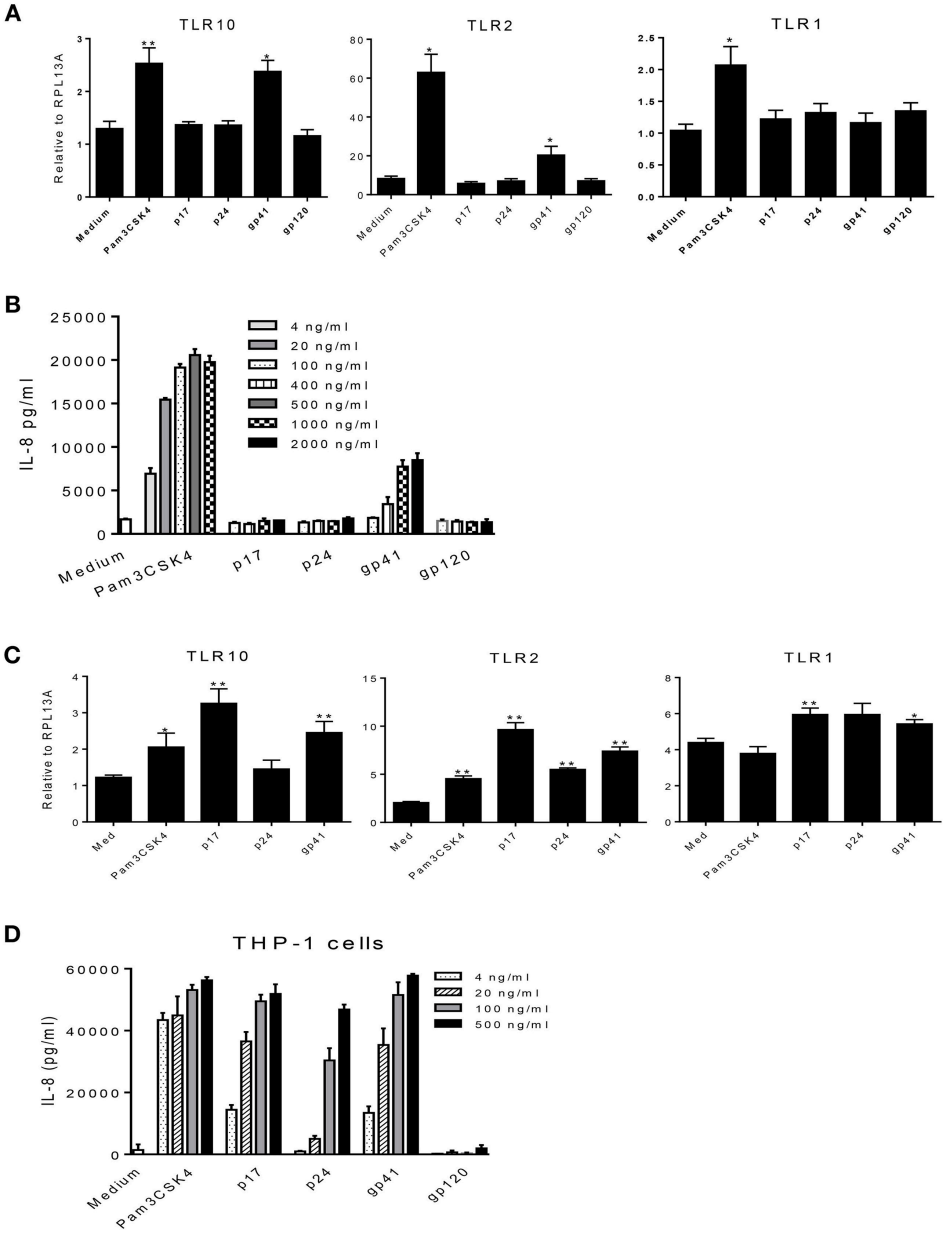
Effects of HIV-1 proteins on TLR expression and cellular responses. **(A)** MCF-10A cells were treated with Pam3CSK4 and the HIV-1 structural proteins: p17, p24, gp41, and gp120. Cellular TLR10, TLR2, and TLR1 mRNAs were analyzed by qRT-PCR. **(B)** IL-8 production in cell culture supernatants in the presence of different concentrations of Pam3CSK4 and HIV-1 proteins were analyzed by ELISA. **(C)** The expression of TLR mRNAs analyzed by qRT-PCR in THP-1 cells treated with Pam3CSK4 and the HIV-1 structural proteins: p17, p24, and gp41. **(D)** IL-8 production in THP-1 cell culture supernatants in the presence of different concentrations of Pam3CSK4 and HIV-1 proteins was analyzed by ELISA. Data set is representative of three different experiments completed in triplicate. Statistic marks: ^*^p < *0.05*, ^**^p < *0.01* for the Mann-Whitney *t*-tests, each group was compared to the medium-treated group (Medium).

HIV-1 structural proteins and glycoproteins have been detected in BM (15) and have been shown to exert immunological effects typical of a proinflammatory response (32–37). Based on our recent findings that HIV-1 structural proteins bind to TLR2 (16), we hypothesized that HIV-1 components may contact the cell surface and trigger TLR10 signaling. Therefore, we next investigated whether specific HIV-1 structural proteins increased cellular activation in primary BM cells. To characterize which HIV-1 structural proteins were involved in this process, we used purified p17 (200 ng/mL), p24 (200 ng/mL), gp41 (1 μg/mL), and gp120 (1 μg/mL) to treat MCF-10A and THP-1 cells and detected the cellular responses. The results demonstrated that the expression of TLR10 and TLR2 mRNA was significantly altered following exposure to gp41 alone compared to untreated medium, as well as Pam3CSK4, which served as a positive control in MCF-10A cells (Figure 5A). IL-8 production increased in a dose-dependent manner in the supernatants of MCF-10A cell cultures containing gp41 and Pam3CSK4 (Figure 5B). However, TLR10 was significantly highly expressed in THP-1 cells in the presence of gp41 as well as p17 (Figure 5C). TLR2 and TLR1 were obviously increased in the presence of gp41, p17, and p24. In the THP-1 cell culture, IL-8 was also correspondingly produced with increases in the amount of Pam3CSK4 and structural proteins, except gp120 (Figure 5D). Together, this data indicates that HIV-1 gp41 is able to induce the innate immune response and alters the level of TLR10 expression, similar to TLR2 (16) and is not cell-type-sensitive.

**Figure 6:**
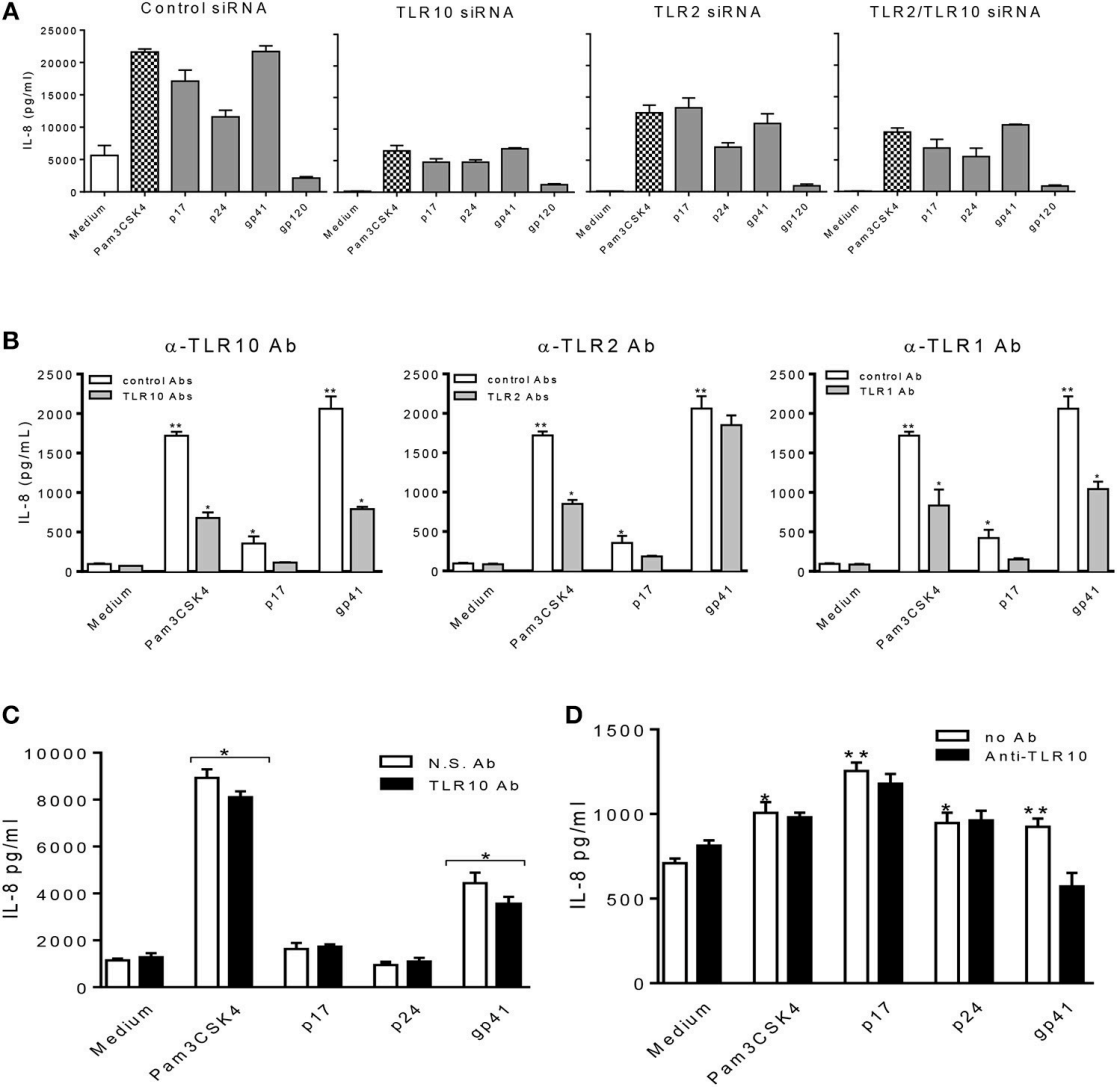
HIV-1 proteins, p17 and gp41 elicit cellular responses through sensing TLR10, TLR2, and TLR1. IL-8 production stimulated by Pam3CSK4, p17, p24, and gp41 was greatly suppressed in THP-1 cells knocked-down of TLR10 with specific siRNA **(A)** and THP-1 cells neutralized with anti-TLR10 antibody **(B)**. **(C)** IL-8 stimulated by Pam3CSK4 and gp41 was reduced in MCF-10A cells neutralized with an anti-TLR10 antibody. **(D)** Primary BM cells displayed a significant decrease in IL-8 production induced by HIV-1 gp41 following neutralization with an anti-TLR10 antibody. Data set is representative of three different experiments completed in triplicate. Statistic marks: ^*^p < *0.05*; ^**^p < *0.01* for Mann Whitney *t*-tests, with each group compared to the medium treated group (Medium).

Furthermore, we confirmed our observations by transfecting THP-1 cells with siRNA specific to target genes (Figure 6A). Compared to the cells treated with the control siRNA, TLR10 siRNA suppressed more than 3-fold of IL-8 production in the cells treated with HIV-1 PAMPs. Treatment with TLR2 siRNA decreased IL-8 production by ~2-fold. The combination of TLR10 and TLR2 siRNAs decreased IL-8 production more than 2-fold. In addition, we used antibodies to neutralize TLR10, TLR2, and TLR1 in gp41 and p17-treated THP-1 cells. As shown in Figure 6B, treatment with anti-TLR10 and TLR1 antibodies significantly reduced IL-8 production in gp41- and p17-treated cells, respectively. Anti-TLR2 antibodies exhibited a similar trend but without a statistical difference. Since the primary focus of this study was TLR10-related events, we only used anti-TLR10 antibodies to treat MCF-10A cells in the neutralization experiments. The results revealed that gp41-induced IL-8 production was also significantly decreased in the presence of the anti-TLR10 antibody (Figure 6C). Similarly, primary BM cells were incubated with the anti-TLR10 antibody before treatment with HIV-1 proteins. The data revealed that gp41-induced IL-8 production was also significantly decreased in the presence of TLR10 antibodies in primary BM cells (Figure 6D). These results suggest that HIV-1 gp41 is capable of upregulating both TLR2 (15) and TLR10 in all cells under our experimental conditions.

### HIV-1 Proteins Induce TLR10, TLR2, and TLR1 Signaling Through the Activation of NF-κBα

**Figure 7:**
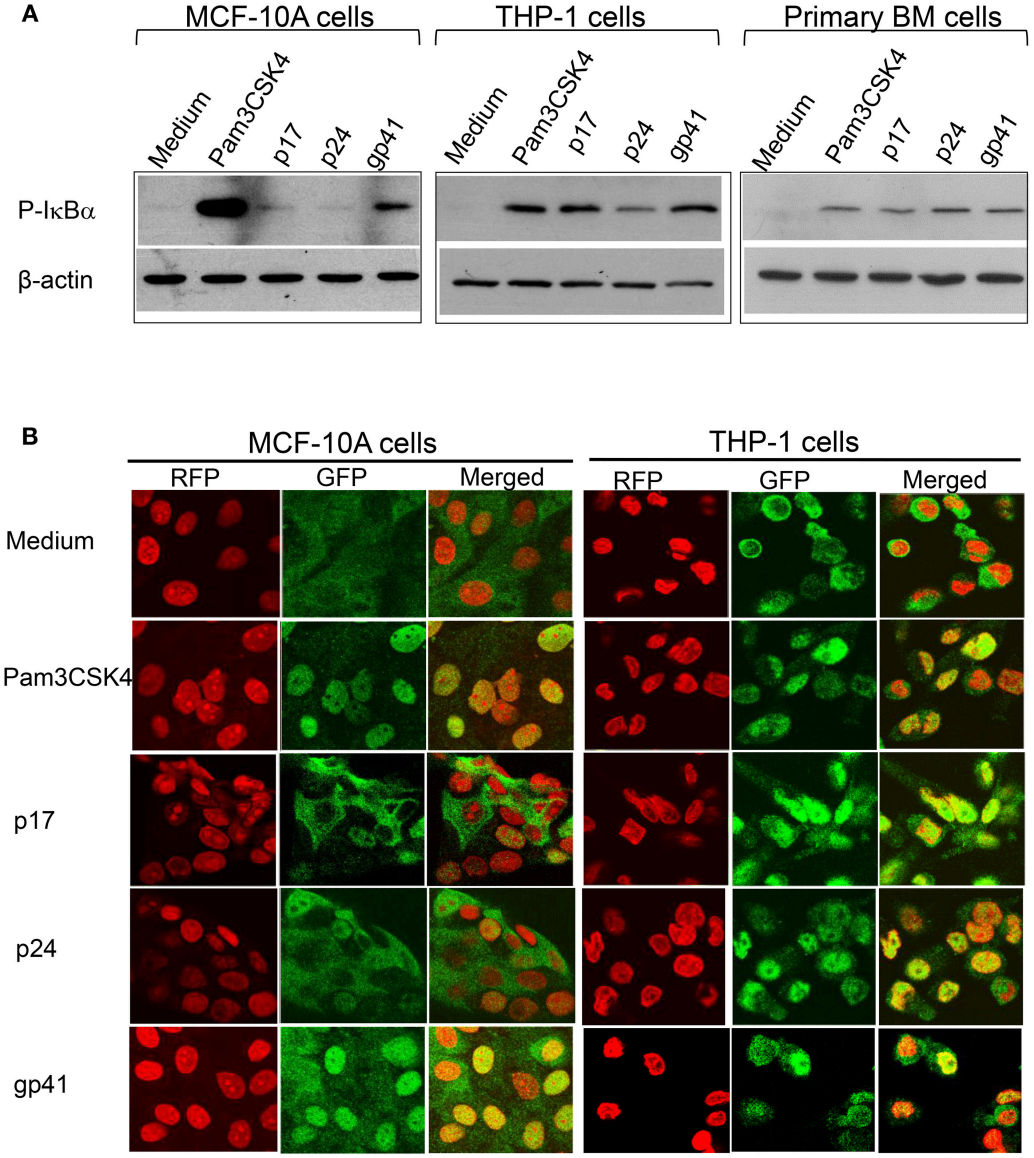
HIV-1 proteins lead to NF-κBα activation **(A)** Western blot analysis of the cell lysates extracted from MCF-10A, THP-1, and primary BM cells treated with HIV-1 proteins and detected with an antibody against the active NF-κBα subunit, phospho(P)-IκBα, with anti β-actin used as the loading control on the same membranes **(B)** Confocal microscopy of HIV-treated MCF-10A and THP-1 cells fixed and stained with an anti- NF-κBα subunit p65 antibody and visualized with a second antibody, anti-rabbit-IgG-alexa488 (GFP; green), p65 was translocated upon activation into the nuclei (RFP; red with propidium iodide staining) displayed in yellow (Merged).

TLR2 and TLR1-mediated signaling pathways have been well-studied. We have recently discovered that the HIV-1 structural proteins p17, p24, and gp41 directly interact with TLR2, inducing NF-κB**α** activation. We then asked whether these structural proteins also induce NF-κB**α** activation via their PAMP effects on TLR10. Cells were treated with Pam3CSK4, p17, p24, and gp41, followed by a Western blot of the lysates with anti-phospho-IκBα, a subunit of NF-κB**α** that is representative of active NF-κB**α**. The results in Figure 7A demonstrate that activated NF-κB**α** was substantially expressed in MCF-10A cells treated with gp41, compared to the positive control (Pam3CSK4) and negative control (medium)-treated cells. In contrast, moderate expression of P-IκBα was observed in the MCF-10A cells treated with p17. However, in both the macrophages derived from THP-1 and BM cells, the three HIV-1 proteins greatly stimulated the production of active NF-κBα. In addition, translocation of the NF-κBα p65 subunit into the nuclei occurred in MCF-10A cells treated with Pam3CSK4 and gp41 (Figure 7B). p65 translocation was observed in a number of MCF-10A cells in the presence of p17 and p24. Compared to the medium control, treatment with Pam3CSK4 and each of the three HIV-1 proteins led to p65 translocation to the nuclei of THP-1 cells.

**Figure 8:**
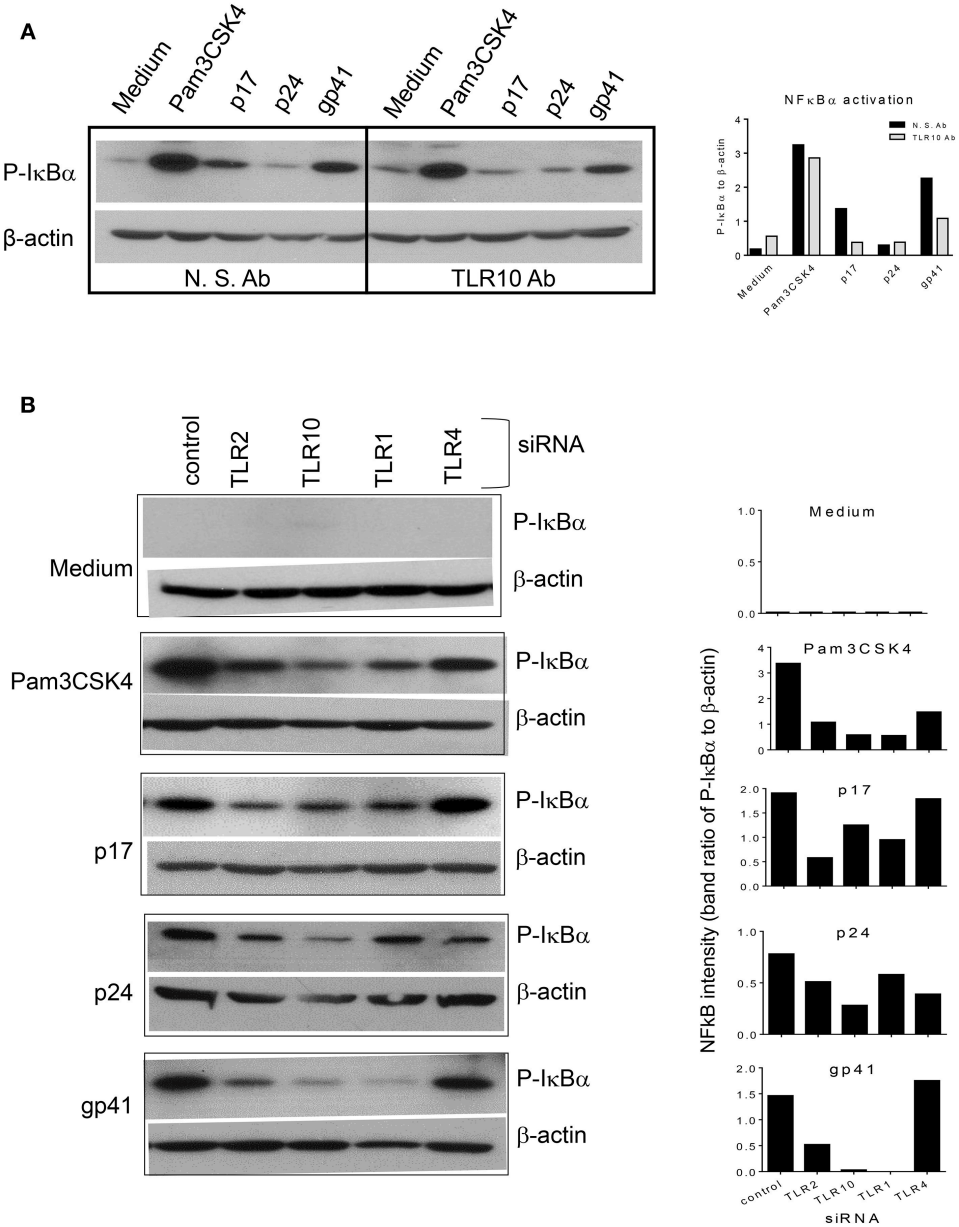
siRNA mediated knockdown or antibody mediated blocking of TLR10 inhibits NF-κBα activation induced by HIV-1 proteins **(A)** anti-TLR10 antibody decreased gp41 induced P-IκBα by half in MCF-10A cells. **(B)** TLR10 siRNA ablated the induction of P-IκBα by gp41 in THP-1 cells.

When TLR10 expression was knocked down using siRNA, NF-κBα activation in response to HIV-1 proteins was significantly reduced in THP-1 cells (Figure 8). The signaling activity induced by the ligation of TLR2 and TLR1 by the positive siRNA control was similar to the response induced by TLR10 activation by the three HIV-1 proteins (p17, p24, and gp41), suggesting that these proteins are involved in the activation of NF-κBα. Considering the possibility that endotoxin was present in the HIV-1 products, we used TLR4 siRNA in the same experiment. The Western blot results clearly showed that knocking down TLR4 did not change the cellular response to p17 and gp41 in activating NF-κBα, which excluded endotoxin or LPS contamination in the two proteins. A 50% reduction in NF-κBα activation was observed in TLR4 siRNA transfected cells treated with Pam3CSK4 and p24, thus indicating cross-talk and a convergence between the different TLR signaling pathways.

## Discussion

To date, a total of 10 (1–10) human TLRs have been identified, which are known to play an important role in the innate immune sensing of invading pathogens (38, 39). Although the ligands for TLRs 1 through 9 are well-known, TLR10 has remained the only orphan receptor with no known ligand, despite being identified >17 years ago (4). To our knowledge, we are the first to report that TLR10 is involved in HIV-1 infection and most importantly, that gp41 acts as a ligand for TLR10 in human macrophages and MECs.

We initially attempted to identify and screen TLRs present in human BM and previously reported that soluble TLR2 (sTLR2) is present in human BM and significantly inhibits HIV-1 infection (15). Furthermore, we also showed that the HIV-1 structural proteins, p17, p24, and gp41, serve as PAMPs for TLR2, leading to the activation of the NF-κBα signaling pathway (16). Surprisingly, with the exception of TLR1 and TLR2, we found significantly higher levels of TLR10 (>100-fold) expression in BM samples collected from HIV-1 infected Nigerian women (Figure 2), thus indicating that TLR10 might play an important role in HIV-1 infection and pathogenesis. In addition, uninfected BM samples from Canadian women exhibited higher basal levels of TLR10 and TLR1 expression compared to uninfected Nigerian women. Although whether there is a significant difference in the level of these two TLRs in healthy populations remains unknown, it is tempting to speculate that the genetic differences present in these two populations might affect the outcome of HIV-1 infection.

TLR1, TLR6, and TLR10 share a common locus on chromosome 4p14 and are structurally similar to each another (40) and are thought to have arisen from several gene duplication events (41). Furthermore, it has been established that TLR10, TLR1, and TLR6 form a single cluster and provide an important first line of defense against infectious agents. Previous work related to TLR1, TLR2, TLR6, and TLR10 has failed to provide the agonist for TLR10 but suggests that the TLR10 ligand might be of a viral origin, leading to the activation of NF-κBα (40, 42). Our current study confirms that one ligand for TLR10 is indeed of viral origin, notably HIV-1 gp41.

TLR10 is known to possess point mutations in its TIR domain that play a critical role in its proper functioning (43). Furthermore, it has been shown that genetic variations in TLR10 can lead to various ailments, such as Crohn's disease (44) and asthma (45). Moreover, TLR10 has been shown to be involved in osteoarthritis (46), Crimean Congo hemorrhagic fever (47), *Listeria monocytogenes, Salmonella* Typhimurium (28), *Helicobacter pylori* (7), and influenza (2). Nevertheless, the involvement of TLR10 in various diseases paves the way for future exploratory studies.

Interestingly, recent data concerning early hominins, Neandertals, and Denisovans, who lived in Europe and Western Asia for over 200,000 years, likely interbred and contributed advantageous genetic alleles to the immune systems of modern humans (48–50). Indeed, TLR genes, which are critical components of innate immunity, have also gained advantageous alleles through this mixing of the gene pool. Notably, adaptive introgression and local positive selection led to the significantly increased expression of the TLR6, TLR1, and TLR10 genes, which facilitated resistance to infectious diseases but may also be associated with increased hypersensitivity to non-pathogenic allergens.

Primary BM cells, MCF-10A MECs, and macrophages display significantly higher levels of TLR10 expression in response to HIV-1. In particular, gp41, not p17, and p24, was able to act as a ligand for TLR10. Since primary BM cells express TLR2, TLR1, and TLR10 (Figures 1, 2); thus, it is plausible that these TLRs could form heterodimers.

Our data have shown that macrophages and MECs are human BM constituents that express TLR1, TLR2, and TLR10. Although gp41 was found to act as a ligand for both TLR2 (16) and TLR10 in the current study. Whether TLR2 and TLR10 interact and associate with each other in the context of an HIV-1 infection remains unclear and will be intriguing to investigate in future studies. However, since macrophages derived from THP-1 cells sensed gp41 in addition to p17 and p24, it is suggested that BM derived macrophages actively participates in HIV-1-induced innate immunity.

Although the induction of TLR10 by immune cells (e.g., B-cells, plasmacytoid dendritic cells, and macrophages) is well-reported (28, 40); the induction of TLR10 by non-immune cells (e.g., intestinal epithelial cells or MECs) raises the possibility of a conserved mechanism regarding TLR10 expression by epithelial cells (28).

We suggest that TLR10 and TLR1 both work cooperatively as the transfection of TRL10 and TLR1 alone or in combination significantly enhanced HIV-1 infection, indicating that TLR10 and TLR1 likely form heterodimers. This is evidenced by the observation that a depletion of TLR10 by a siRNA-mediated knockdown negatively impacted the proviral integration of HIV-1. Moreover, TLR10 knockdown led to significantly lower levels of IL-8 production in cells treated with gp41, p17, and p24. Furthermore, gp41, p17, and p24 were able to activate NF-κBα, which was readily blocked using an anti-TLR10 antibody or TLR10-specific siRNA-treated cells, suggesting that TLR10 signaling led to the activation of NF-κBα and ultimately the production of IL-8.

Importantly, the present study provides the first evidence that HIV-1 gp41 functions as a ligand for TLR10 in MCF-10A and THP-1 cells. Recently, double-stranded RNA (dsRNA) has been shown to act as a ligand for TLR10 (51). In addition, the same study demonstrated that TLR10 competes with TLR3 for binding to ligands in various endocytic pathways, ultimately leading to the induction of interferons. Similarly, synergy between TLR3 and TLR10 has been suggested in the context of an influenza virus infection. Although our current data demonstrates the involvement of TLR10 in HIV-1 infection, the existence of such synergy between TLR10 and TLR3 in an HIV-1 infection cannot be dismissed and requires further future investigation.

Our data demonstrated that HIV-1 gp41 acts as a ligand for TLR10 and leads to the induction of IL-8 and NF-κBα activation. However, our observation is contradictory to some previous reports where TLR10 expression has been shown to suppress the cytokine responses (1, 52). These contradictions could be due to differences in cell-type use, ligand specificity or geographical location. Further, divergent roles of TLR10 have been suggested to be due to the mechanism of cross-talking between TLR10 and TLR3 (51) or perhaps the interaction of TLR10 with TLR2, TLR1 and TLR6 (1). Although, the exact mechanism is not known; we cannot exclude the possibility of spontaneous heterodimerization of TLR2 and TLR10 (1) due to HIV-1 gp41 which acts as a ligand for both TLR2 (16) and TLR10.

The present investigation demonstrated that TLR10 acts as an innate immune sensor for HIV-1 infection and leads to the induction of IL-8. Furthermore, we showed that blocking TLR10 with either an anti-TLR10 antibody or siRNA attenuates gp41-mediated NF-κBα activation (Figure 8). Interestingly, TLR1 depletion through siRNA knockdown also inhibited gp41-mediated NF-κB activation, suggesting that TLR1 and TLR10 might form a heterodimer. Recent evidence shows that TLR10 plays a key role in the innate immune response following an influenza infection (2). Specifically, it was found that silencing of the TLR10 gene impacted IL-8 production in human influenza virus-infected macrophages. Similarly, we observed that when TRL10 is silenced in HIV-1 infected macrophages, IL-8 production is severely impaired, thus raising the possibility that there is a conserved mechanism during HIV-1 and influenza virus infections in human macrophages.

Our studies have shown that human primary BM cells express significantly higher levels of TLR1, TLR2, and TLR10. Currently, the precise role of these TLRs in infants following BM ingestion remains unknown. However, in our other ongoing studies, we have identified some novel exosomal miRNAs in BM samples exclusive to HIV-1 infection present in mother's milk (data not shown) that could potentially play a protective role during MTCT of HIV-1 in conjunction with these TLRs.

Taken together, our data reveal for the first time that TLR10 is involved in HIV-1 infection. Furthermore, gp41 was found to act as a novel ligand for TLR10. We believe that the data shown in the present study describing an HIV-1 ligand for TLR10 will advance our current knowledge, as well as further our understanding of the role of TLR10 in innate immunity during HIV-1 infection.

## Materials and Methods

### Human Subjects

HIV-1-uninfected (Hamilton, Ontario, Canada and Nigeria) and HIV-infected (Nigeria) women were recruited to participate in the present study. All HIV-infected women were in the chronic phase of infection as determined by at least two positive HIV-1 serological tests separated by at least 1 year. All women were sampled during voluntary “healthy” research visits as per the cohort protocol, and therefore, were not acutely ill at the time of assessment. Written as well as informed consent for the collection of demographics, behavioral data and biological samples were obtained from all study participants. Inclusion criteria included breastfeeding women who were HIV-uninfected and did not report complications *in utero* during their full-term pregnancies or intrapartum. Women were excluded if they had cesarean sections, their pregnancies were not full-term, or they were diagnosed with mastitis post-partum. Samples included in these analyses were obtained from women who were not taking medications other than vitamin supplements intra- or post-partum and did not receive an epidural intra-partum. Three mothers reported minor colic and one reported silent reflux in their infants. The study was approved by the institutional review boards of the University of Manitoba Hospital ethical review committee. All clinical investigations were conducted according to the principles of the Helsinki Declaration. The study was approved by the McMaster Research Ethics Board (REB Approval #08-176) and the CCI of Children's Hospital, Los Angeles.

### Sample Acquisition and Preparation

Milk samples were self-collected into sterile tubes within the first week and at 1, 3, and 6 months post-partum, and immediately shipped on ice for processing in our laboratory. The samples were separated into lipid, supernatant, and cellular fractions and stored at −80°C and liquid nitrogen, respectively. BM supernatant fractions were used for Western blotting or were filter-sterilized (0.45 μm) for functional assays to avoid cellular contamination.

### Cells and Reagents

THP-1 and primary BM cells were cultured in RMPI-1640 supplemented with 10% FBS, 2 mM L-glutamine, 10 mM HEPES, 1 mM Sodium Pyruvate, and 1% penicillin/streptomycin (Invitrogen). MCF-10A cells (American Type Culture Collection, Manassas, VA) were maintained in Dulbecco's modified Eagle's medium-F12 (DMEM/F12, Invitrogen) supplemented with 5% horse serum (Invitrogen), 100 ng/mL cholera toxin (Sigma), 10 μg/mL insulin (Sigma), 20 ng/mL recombinant human EGF (Peprotech), 0.5 μg/mL hydrocortisone (Sigma), and 1% penicillin/streptomycin (Invitrogen). The TZMbl cells were cultured in DMEM supplemented with 10% FBS and 1% (vol/vol) each of l-glutamine, penicillin, and streptomycin with and without 0.8 mg/mL geneticin (G418; Invitrogen, Burlington, ON, Canada), respectively, as described previously (16, 29). HIV-1 proteins included p17 (Virogen, Mississauga, ON, Canada), p24, and gp41 (Genway Biotech, Inc., San Diego, CA, USA). TLR ligands, ssRNA40 (Mobix, McMaster University), LPS, Pam3CSK4 (InvivoGen), and poly I:C (Sigma-Aldrich, Burlington, ON, Canada) were diluted in phosphate-buffered saline (PBS).

### Quantitative Reverse-Transcriptase Real-Time Polymerase Chain Reaction

Total RNA was extracted from the cells using Trizol as per the manufacturer (Invitrogen, Carlsbad, California, USA) and treated with DNase I, DNA-free (Ambion, Austin, Texas, USA). Reverse transcription (RT) reactions and quantitative real-time PCR (qRT-PCR) was performed as previously described (11).

### Small Interfering RNA Knockdown

siRNA molecules targeting human TLR1 (Sigma-Aldrich, SASI_Hs01_00162170/AS), TLR2 (Sigma-Aldrich, SASI_Hs01_ 00081589/AS), TLR4 (Sigma-Aldrich, SASI_Hs01_00122250/AS), TLR10 (Sigma-Aldrich, SASI_Hs01_00099892), or non-targeting siRNA (Invitrogen, 129201 H07/129296 H05) were purchased and used as previously described (16). The cells were exposed to specific HIV-1 proteins, and the supernatants were collected to quantify the level of pro-inflammatory cytokine production as previously described (16).

### Flow Cytometry

Flow cytometry analysis was performed as previously described (53). Briefly, 10 mL of fresh healthy human BM containing ~5 × 10^6^ cells collected within 3 months of lactation, was centrifuged at 2,000 × g at room temperature for 10 min, washed with PBS, and centrifuged two more times. The cells were first incubated with Live/Dead Fixable Aqua (ThermoFisher) at room temperature for 30 min and subsequently washed twice with PBS. Besides compensation and FMO controls, two sets of cells were immune-stained at room temperature for 30 min in 0.5% BSA-PBS containing a combination of conjugated antibodies (CD45-PerCP-Cy5.5 BD, CD14-V450 BD, MUC1-PE-Cy7 BioLegend, TLR2-APC R&D Systems, TLR10-PE BioLegend) and (CD45-PerCP-Cy5.5, CD14-V450, MUC1-PE-Cy7, TLR1-APC R&D Systems, TLR10-PE), respectively. The cells were washed twice with PBS and fixed with 1% PFA-PBS. The samples were run on a BD LSRFortessa and analyzed using FlowJo software.

### Establishment of a Stable TLR10 Reporter Cell Line

The TLR10 expression plasmid, pIhT10G, was subcloned from pUNO1-hTLR10 (InvivoGen) into pIRES2-Green1 vector (Clontech), with a *Sac*I-*BamH*I fragment via PCR using Phusion DNA polymerase (New England Biolabs, USA). Transfection with 2.5 μg pIhT10G and 5 μL Lipofectamine 2,000 into TZMbl cells was performed in a six-well plate for 2 days. The cells were trypsinized and re-suspended in 0.2–1.4 mg/mL series of G418 geneticin (Thermo Fisher Scientific, MA, USA) containing growth medium in a 96-well plate, and cultured for at least 7 days, followed by repeating the selection for a total of three times in the same manner. The established cells were named TZMbl-10 and cultured in 0.2–0.4 mg/mL geneticin growth medium.

### HIV-1 Infection and Integration

TZMbl and the derived TZMbl-2 and TZMbl-10 cells were transfected with plasmids, phT2G (16), phT10G, and pUNO1-hT1 (InvivoGen) expressing TLR10, TLR2, and TLR1, with PolyJet (SignaGen Rockville, MD, USA) in a 96-well plate for 24 h. The cells were infected with HIV-1 BAL 30-50 TCID_50_/96-well plus 12 μg/mL DEAE-dextran for 45 h for HIV-1 infection. The proviral DNA analysis was performed by measuring the relative light units of luciferase activity for 8 h.

THP-1 cells grown in a 96-well plate were expanded in 8 mM PMA-growth medium for 3 days and PMA-free medium for another 2 days. The cells were then transfected with specific siRNAs using Lipofectamine RNAiMAX reagent (Thermo Fisher Scientific, MA, USA) for 2 days. The cells were infected with HIV-1 BAL 100 TCID_50_/well plus 4 μg/mL polybrene overnight and washed with growth medium and cultured for a total of 7 days. HIV-1 integration was detected by the isolation of total cellular DNA using proteinase K digestion and phenol extraction, followed by PCR of the viral pol gene. The proviral DNA was normalized to the cellular 18S rRNA gene in a same sample.

### IL-8 ELISA

IL-8 production from the cell culture was assessed via an EB ELISA in 20–100-fold dilution as per the manufacturer's instruction as described previously (16).

### Stimulation of Primary BM Cells *in vitro* and Neutralization

Healthy BM was diluted 2-fold in PBS and the cells were isolated by centrifugation at 800 × g for 20 min. The lipid layer and fluid were removed, and the cell pellet was washed twice with PBS. The cells were resuspended in a 96-well plate at a density of 1.5 × 10^5^ per well with RPMI-1640 medium and cultured overnight.

Since none of the available anti-TLR10 antibodies were able to efficiently neutralize TLR10 alone under our experimental conditions (data not shown), we used a cocktail of TLR10 antibodies from different sources comprising rabbit polyclonal sc-30198 (Santa Cruz Biotechnology, Santa Cruz, CA, USA), mouse monoclonal sc-293300 (Santa Cruz Biotechnology, Santa Cruz, CA, USA), mouse anti-human TLR10 H00081793-M01 (Novus Biologicals), and mouse monoclonal MA6619 (R&D Systems) compared to a cocktail control which consisted of rabbit and mouse isotype IgG controls (Santa Cruz Biotechnology, Santa Cruz, CA, USA).

### Western Blot

The cells were washed with PBS and the lysates were prepared as previously described (16). Protein quantification was performed using a DC^TM^ protein assay kit (Bio-Rad) and run on SDS-PAGE gels. The primary antibodies consisted of anti-TLR1, anti-TLR2 (R&D Systems), anti-TLR4 (Novus Biologicals), anti-TLR10 (Santa Cruz Biotechnology, Santa Cruz, CA, USA), phospho-IκBα #9246 (Cell Signaling), NF-κBα p65 sc-372 (Santa Cruz Biotechnology, Santa Cruz, CA, USA), and anti-β-actin antibodies. HRP-labeled goat anti-mouse IgG 1706516 (Bio-Rad) and HRP-labeled goat anti-rabbit IgG 1706515 (Bio-Rad) were used as secondary antibodies.

### Confocal Microscopy

Immunofluorescence staining was performed as previously described (54). MCF-10A and THP1 cells grown on an eight-well BD Falcon culture slides (BD Biosciences) were either left untreated or treated with Pam3CSK4, or the HIV-1 proteins p17, p24, and gp41. The cells were fixed and immune-stained with NF-κBα p65 (1:500) to detect the nuclear translocation of NF-κBα p65. Corresponding Alexa Fluor 488 conjugated IgG (Molecular Probes, Eugene, OR, USA) was used as a secondary antibody. The images were acquired using an inverted laser-scanning confocal microscope (LSM 510, Zeiss, Oberkochen, Germany).

### Statistical Analysis

The data were plotted and analyzed using Microsoft Excel, SPSS, and Prism 4.0 software and shown as Mean ± SEM (GraphPad Software, San Diego, California, USA). Non-parametric tests were used, including Mann-Whitney U-tests for unmatched comparisons, Wilcoxon paired *t*-tests for matched samples, and a Spearman's rank test for matched correlations.

## Data Availability

All datasets generated for this study are included in the manuscript and/or the supplementary files.

## Author Contributions

BH, X-YD and MZ performed the experiments and wrote the manuscript. AA and SO performed clinical studies and collected the human samples. BH and KR conceived the idea. KR supervised the work, obtained the grant and wrote the manuscript.

### Conflict of Interest Statement

BH is an employee of Evolve Biosystems. The remaining authors declare that the research was conducted in the absence of any commercial or financial relationships that could be construed as a potential conflict of interest.

## Supplementary Material

Supplementary Figure 1 Confirmation of the siRNA mediated knockdown of TLR2, TLR10, TLR1, and TLR4 in THP-1 cells by Western blot. THP1 cells were either transfected with the specific siRNA targeting the respective TLRs or with a scrambled control. Lysates were prepared after 48 h and subjected to Western blot analysis using the indicated antibodies.
