# Supplementary figures and images for: TLR10 Senses HIV-1 Proteins and Significantly Enhances HIV-1 Infection
Source: Front Immunol. 2019 Mar 15;10:482. doi: 10.3389/fimmu.2019.00482 (PMC6430187; doi:10.3389/fimmu.2019.00482)

# Supplementary Figure 1

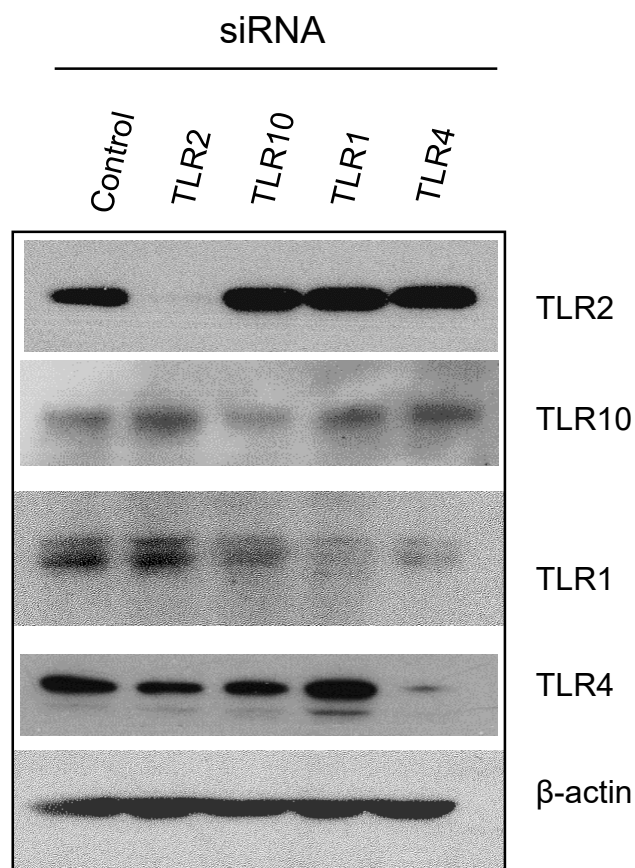

Supplement: Supplementary Figure 1 — Confirmation of the siRNA mediated knockdown of TLR2, TLR10, TLR1, and TLR4 in THP-1 cells by Western blot. THP1 cells were either transfected with the specific siRNA targeting the respective TLRs or with a scrambled control. Lysates were prepared after 48 h and subjected to Western blot analysis using the indicated antibodies. [file Image_1.pdf]
